# Proteomic Analysis of Cerebrospinal Fluid in a Fulminant Case of Multiple Sclerosis

**DOI:** 10.3390/ijms13067676

**Published:** 2012-06-21

**Authors:** Judit Füvesi, Jörg Hanrieder, Krisztina Bencsik, Cecilia Rajda, S. Krisztián Kovács, László Kaizer, Sándor Beniczky, László Vécsei, Jonas Bergquist

**Affiliations:** 1 Department of Neurology, Albert Szent-Györgyi Clinical Center, University of Szeged, Szeged 6725, Hungary; E-Mails: fuvesi.judit.katalin@med.u-szeged.hu (J.F.); bencsik.krisztina@med.u-szeged.hu (K.B.); rajda.cecilia@med.u-szeged.hu (C.R.); beniczky.sandor@med.u-szeged.hu (S.B.); vecsei.laszlo@med.u-szeged.hu (L.V.); 2 Analytical Chemistry, Department of Chemistry-Biomedical Center, Uppsala University, P.O. Box 599, Uppsala 75124, Sweden; E-Mail: jorg.hanrieder@chalmers.se; 3 Analytical Chemistry, Department of Chemical and Biological Engineering, Chalmers University of Technology, Kemivägen 10, Göteborg 41296, Sweden; 4 Department of Clinical Neurophysiology, Danish Epilepsy Centre, Dianalund 4293, Denmark; 5 Department of Pathology, Albert Szent-Györgyi Clinical Center, University of Szeged, Szeged 6725, Hungary; E-Mails: kidjo@freemail.hu (S.K.K.); kaizer.laszlo@med.u-szeged.hu (L.K.); 6 Neuroscience Research Group, Hungarian Academy of Sciences, University of Szeged, Semmelweis str 6, Szeged 6725, Hungary; 7 Science for Life Laboratory (SciLife Lab), Uppsala University, P.O. Box 599, Uppsala 75124, Sweden

**Keywords:** multiple sclerosis, fulminant, shotgun-proteomics, protein quantification, isobaric tag labeling, matrix assisted laser desorption/ionization time of flight tandem mass spectrometry (MALDI TOF/TOF MS)

## Abstract

Multiple Sclerosis (MS) is a chronic disease, but in rare fulminant cases rapid progression may lead to death shortly after diagnosis. Currently there is no diagnostic test to predict disease course. The aim of this study was to identify potential biomarkers/proteins related to rapid progression. We present the case history of a 15-year-old male MS patient. Cerebrospinal fluid (CSF) was taken at diagnosis and at the time of rapid progression leading to the patient’s death. Using isobaric tag labeling and nanoflow liquid chromatography in conjunction with matrix assisted laser desorption/ionization time of flight tandem mass spectrometry we quantitatively analyzed the protein content of two CSF samples from the patient with fulminant MS as well as one relapsing-remitting (RR) MS patient and one control headache patient, whose CSF analysis was normal. Seventy-eight proteins were identified and seven proteins were found to be more abundant in both fulminant MS samples but not in the RR MS sample compared to the control. These proteins are involved in the immune response, blood coagulation, cell proliferation and cell adhesion. In conclusion, in this pilot study we were able to show differences in the CSF proteome of a rapidly progressing MS patient compared to a more typical clinical form of MS and a control subject.

## 1. Introduction

Multiple sclerosis is a demyelinative disorder of the central nervous system (CNS). The onset of the disease is in young adulthood in most cases, and although it usually does not decrease life expectancy it has a great impact on quality of life. As it affects patients in the most active period of their life it has a substantial socioeconomic burden as well.

The etiology of the disease is still unknown. The typical disease course starts with a relapsing-remitting (RR) phase, with even complete remissions at the beginning and a gradual accumulation of residual symptoms over time. After a variable period of time (years-decades) the disease turns into a secondary progressive form characterized by a continuous progression with or without relapses. In 10–15 percent of cases the disease is progressive from the very beginning: primary progressive form. In very rare cases the disease course is fulminant and rapid progression leads to death shortly after diagnosis.

Currently there is no available diagnostic test to predict disease course and prognosis in individual patients, therefore identification of biomarkers that at an early stage can differentiate between clinical forms and predict severity of the disease is of great importance.

Over the last decade, proteomics has proven to be a powerful approach to gain insight into biological processes by monitoring the whole protein content of a certain sample at a distinct point of time. The major challenge in proteome analysis of complex biological samples like body fluids, such as plasma, cerebrospinal fluid (CSF) or urine, is to deal with the large dynamic range of the abundant proteins. Therefore, high resolving protein/peptide separation methods in combination with very accurate and high resolving mass spectrometric tools are essential for sensitive and significant protein identification [1–4]. However, differences in protein expression in clinical studies and their correlation to the disease are the main focus of interest [5]. Quantitative proteomic methods, like isobaric tag labeling, provide information about relative protein concentrations [6,7] and also allow for multiplexed approaches.

The aim of this study was to identify potential protein biomarkers in CSF related to rapid progression in multiple sclerosis using a differential shotgun-proteomic approach based on isobaric tag labeling and nanoLC-MALDI TOF/TOF MS. Although in recent years a number of papers appeared on proteomics of multiple sclerosis CSF, this is to the best of our knowledge the first report of a fulminant case combined with proteomic analysis of CSF.

## 2. Results

### 2.1. Experimental Design for MS Analysis

**Figure 1:**
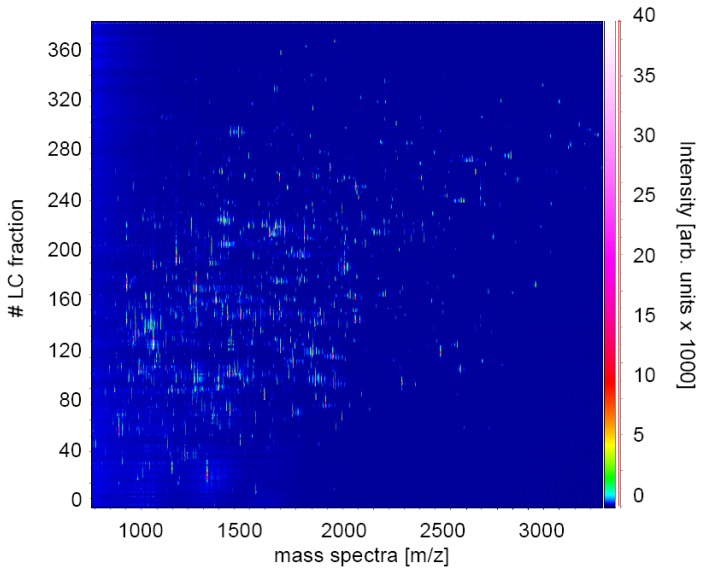
Mass chromatogram of one LC-MALDI experiment illustrating separation efficiency and hence improved sample complexity reduction. Two-dimensional survey of all TOF-MS spectra (*x*-axis) acquired from each collected fraction during peptide elution (*y*-axis, number of spectra and LC fractions, respectively). The values on the *z*-axis (rainbow-scale) indicate mass peak intensities in arbitrary units.

The presented bottom up-strategy, based on isobaric tag labeling in conjunction with enzymatic digestion followed by nanoLC coupled off-line to MALDI TOF/TOF MS, has proven to be a powerful proteomic approach for quantitative protein profiling in complex biological samples. A satisfactory distribution of contingent precursor candidates for subsequent MS/MS experiments was achieved using the described protocol, which is essential for increasing sensitivity and thereby to enhance the protein identification rate (Figure 1).

### 2.2. Protein-Identification by Combined Database Search

The acquired LC-MS/MS data were combined in one data file and subjected to comprehensive database search following the mascot algorithm (www.matrixscience.com). Each protein was considered to be a positive match if it was identified by at least one MS/MS that fulfilled criteria of significance. The significance threshold was set to at least 99% (*p* < 0.01) and peptide uniqueness was required indicating identity or extensive homology.

We have identified 78 proteins in the samples with a false positive identification rate of <2% (Supplementary Table S1). Among the findings, acute phase reactants and a large number of highly abundant proteins like albumin, immunoglobulins, apolipoproteins, hemoglobins, haptoglobin and transferrin were detected. Furthermore, some less abundant brain derived proteins including Limbic-system associated membrane protein and Neural cell adhesion molecule 1 were also identified. The findings further include transport proteins, immunoglobulins, glycoproteins, coagulation factors, complement factors, enzymes, inhibitors and structural/membrane-associated proteins.

In order to validate the observed quantitative data, technical and biological replicate experiments were performed. Biological replicates included repeated processing (digestion and isobaric tag labeling) and analysis of the clinical samples. Technical replicates included repeated analysis of processed sample in order to evaluate precision of the technical platform. Here relative standard deviation values of 1%–23% for biological replicate experiments and values of 3%–8% for technical variation were observed [8].

### 2.3. Differential Protein Profiles

Isobaric tag labeling by means of iTRAQ was utilized for quantitative protein profiling. The main advantage of this approach is that the samples are analyzed under exactly the same conditions and the quantification is performed in the MS/MS mode. This limits the risk of systematic errors, increases the signal-to noise ratio (S/N) and results in a high reliability of the obtained data. Furthermore, a high sample throughput can be achieved because multiple samples are processed in parallel [7].

**Figure 2:**
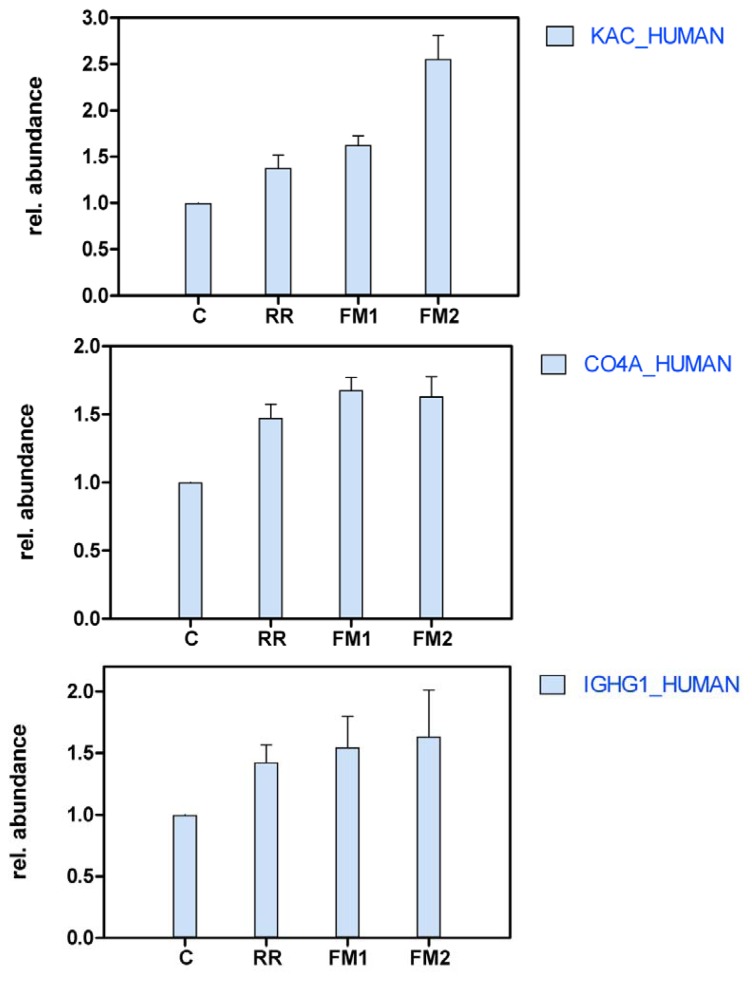

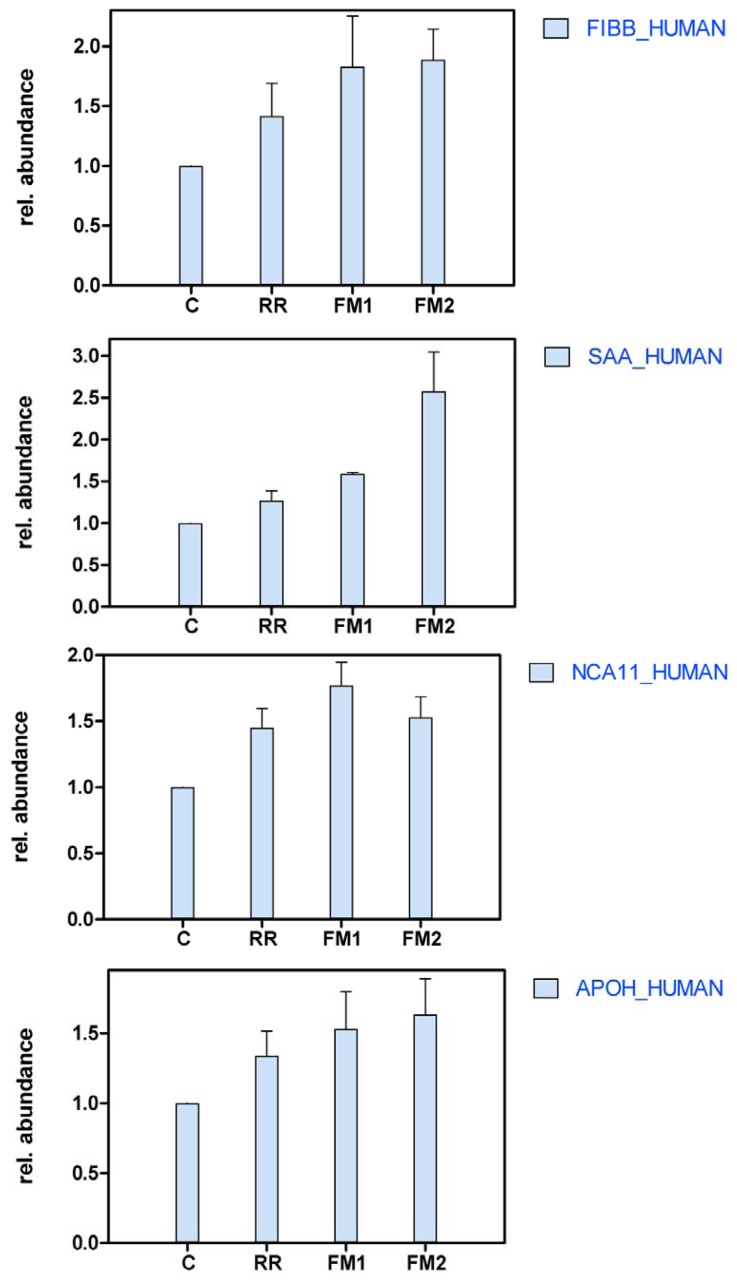
Proteins more abundant in both fulminant Multiple Sclerosis (MS) samples but not in relapsing-remitting (RR) MS compared to control: Ig kappa chain C region (KAC). Complement C4-A (CO4A). Ig gamma-1 chain C region (IGHG1). Fibrinogen beta chain (FIBB). Serum amyloid A protein (SAA). Neural cell adhesion molecule 1 (NCA11). Beta-2-glycoprotein 1 (APOH). Error bars represent the standard deviation (*n* = number of significantly identified peptides that provide significant quantitative information).

Thirty proteins were found to be increased in at least one MS sample compared to the control sample (Table 1). Three proteins, hemoglobin alpha, beta and delta chains, were decreased in the multiple sclerosis samples compared to the headache patient control. This could potentially reflect a minor contamination due to puncture bleeding in the headache patient, but there were no visible signs of such contamination. In case of the remaining 48 proteins, we found no difference in abundance (Table 1, Supplementary Table S1). The biggest increase in abundance was observed for the following proteins: Ig kappa chain C region (IgK) (sample FM2), Osteopontin (OSTP) (FM2), Serum amyloid A protein (SAA) (FM2), Basement membrane-specific heparan sulfate proteoglycan core protein (PGBM) (FM1). Seven proteins were found to be up-regulated in both fulminant MS samples but not in the relapsing-remitting case compared to the control. These proteins included Ig kappa and gamma-1 chain C region, Complement C4-A, Fibrinogen beta chain, Serum amyloid A protein, Neural cell adhesion molecule 1 and Beta-2-glycoprotein 1 (Figure 2).

Ig kappa chain C region and Ig gamma-1 chain C region are proteins making up the constant regions of immunoglobulins [9]. This region is invariable in its amino acid sequence within any class of immunoglobulin.

Complement C4 has a central role in the activation of the classical pathway of the complement system [9]. Activated C1 removes the C4a anaphylatoxin from the alpha chain. It is a mediator of local inflammatory process, induces the contraction of smooth muscle, increases vascular permeability and causes histamine release from mast cells and basophilic leucocytes. Also, it is a blood group antigen.

Fibrinogen contains 2 sets of 3 non-identical chains (alpha, beta, and gamma) [9]. In the process of blood coagulation, conversion of fibrinogen to fibrin is triggered by thrombin, which cleaves fibrinopeptides A and B from alpha and beta chains.

Beta-2-glycoprotein 1 binds to various negatively charged substances, such as heparin and phospholipids. It may prevent the activation of the intrinsic blood coagulation cascade by binding to phospholipids on the surface of damaged cells.

Serum amyloid A protein is a major acute phase reactant. In reactive, secondary amyloidosis SAA protein accumulates in the extracellular space of various tissues. These deposits alter tissue structure and interfere with normal function.

Neural cell adhesion molecule 1 is a cell membrane protein [9]. It is involved in neuron-neuron adhesion, neurite fasciculation and outgrowth of neurites.

## 3. Discussion

In this report we present the application of a rapid, quantitative proteomic analysis for identifying differentially expressed proteins in the CSF of a fulminant multiple sclerosis patient and control samples.

In recent years a number of papers appeared describing proteomic analysis of CSF or brain tissue of multiple sclerosis patients [10–14]. The clinical characteristics of the samples as well as the methods used in these studies are rather diverse, making their comparison very difficult.

Hammack *et al*. [10] reported the analysis of a pooled sample of three relapsing-remitting MS patients and a pooled sample of three patients with non-MS inflammatory CNS disorders using two-dimensional gel electrophoresis and peptide mass fingerprinting. They identified four proteins in the gels containing MS CSF that were not reported previously in normal human CSF: CRTAC-1B (cartilage acidic protein), tetranectin (a plasminogen-binding protein), SPARC-like protein (a calcium binding cell signalling glycoprotein) and autotaxin t (a phosphodiesterase). Among these, tetranectin and SPARC-like protein were identified in our samples as well, and were found to be elevated in the RR and the FM1 sample.

In the study of Dumont *et al*. [11] CSF samples from five MS patients (4 RR, one secondary progressive) were analyzed by two-dimensional gel electrophoresis followed by liquid chromatography tandem mass spectrometry. With this method 15 proteins have been identified that were not previously observed on non-multiple sclerosis CSF 2-DE gels. These proteins were: psoriasin, calmodulin-related protein NB-1, annexin 1, EWI-2, Niemann-Pick disease type C2 protein (NPC-2), semenogelin 1 (SEM1), semenogelin 2 (SEM2), complement factor H-related protein 1 (FHR-1), procollagen C-proteinase enhancer protein (PCPE), aldolase A, *N*-acetyllactosaminide β-1,3-*N*-acetylglucosaminyl-transferase, tetranectin, cystatin A, superoxide dismutase 3 and glutathione peroxidase (Supplementary Table S1).

Lehmensiek *et al*. [12] compared CSF samples from RR MS and clinically isolated syndrome (CIS) patients with controls using two-dimensional difference gel electrophoresis (2-D-DIGE) and MALDI-TOF mass spectrometry. In RR MS Ig kappa chain NIG93 protein was increased in concentration, while transferrin isoforms, alpha 1 antitrypsin isoforms, alpha 2-HS glycoprotein, Apo E and transthyretin decreased. In our study transferrin was found to be more abundant in the FM1 sample but not in the other fulminant and the RR sample. Apo E showed the same pattern. In contrast, transthyretin showed no change compared to the control.

In a recently published study of Stoop *et al*. [13] significant differences were observed comparing the peak lists of spectra from CSF of MS patients and patients with other neurological diseases (OND), and also clinically isolated syndrome (CIS) *vs*. OND. Three differentially expressed proteins were identified in the CSF of MS patients compared to CSF of patients with OND: chromogranin A, clusterin and complement C3.

The same group compared proteome profiles of CSF from RR and primary progressive (PP) multiple sclerosis and found that they overlap to a large extent [15]. The main detected difference was that protein jagged-1 was less abundant in PP MS compared to RR MS, while vitamin D-binding protein was only detected in the RR MS CSF samples. Ottervald *et al*. found an increased CSF level of vitamin-D-binding protein in SP MS compared to the control [16]. In our samples this protein was detected, but there was no significant difference across the samples, probably due to the fact that we did not analyze CSF from progressive clinical forms of the disease. Recently, impaired vitamin D homeostasis has been linked to multiple sclerosis [17]: high serum levels of 25-hydroxyvitamin D correlated with a reduced risk of MS [18] and Vitamin D supplementation was proposed as an add-on therapy [19].

In our study among the seven proteins found to be more abundant in fulminant MS, fibrinogen and β-2-glycoprotein 1 are involved in the process of blood coagulation. Interestingly, in a study examining the proteomics of multiple sclerosis lesions Han *et al*. [14] identified several proteins involved in coagulation that were uniquely found in chronic active plaque samples: tissue factor, protein C inhibitor, thrombospondin, fibronectin and vitronectin. This finding suggested dysregulation of molecules associated with coagulation. In further experiments both tissue factor and activated protein C administration ameliorated disease severity in experimental autoimmune encephalomyelitis (EAE), an animal model of multiple sclerosis.

Fibrinogen, which was found to be up-regulated in our study in the CSF of the fulminant MS patient, was shown to be deposited perivascularly in MS plaques [20]. Blood-brain-barrier disruption, a common feature in MS lesions permits the leakage of fibrinogen into the CNS. Fibrinogen induces the differentiation of microglia to phagocytes via the integrin receptor Mac-1 [20]. Microglia activation leads to phagocytosis and tissue damage in inflammatory demyelination. In the study of Adams *et al*. a fibrin-derived γ^377–395^ peptide was able to inhibit fibrinogen-Mac-1 interaction and microglia activation without affecting the procoagulant properties of fibrinogen [20]. The peptide was found to ameliorate symptoms of mice with EAE, therefore it may be a novel therapeutic strategy for MS.

Serum amyloid A protein had an elevated level in both fulminant samples. In earlier studies it was shown to be elevated in the serum of RR multiple sclerosis patients [21], and also a transient increase of SAA was observed after intramuscular interferon-β 1a administration [22].

From the up-regulated proteins in fulminant MS, immunoglobulin and complement proteins take part in inflammation and immune response. Their elevated level in CSF may account for the rapid progression of the disease.

In histopathological studies, multiple sclerosis lesions are grouped in four types based on immunocytochemical characteristics of demyelination patterns [23]. Pattern II lesions are characterized by antibody-complement mediated damage. Considering our case, there are regions showing characteristics of pattern II demyelination. In a recent immunohistochemical study Mahad *et al*. [24] analyzed mitochondrial respiratory chain proteins in active lesions from acute fulminant multiple sclerosis (pattern II and pattern III lesions) and white matter stroke cases containing lesions with active demyelination. In pattern II lesions in the early active stage they observed perivenous sheaths of demyelination separated by areas of partly preserved myelin with abundant macrophages, containing early myelin degradation products. At higher magnification, the tissue in early active and late active stages appears vacuolated, due to profound edema, which extends into the adjacent normal white matter. This histological description (and also the slides in the paper) is strikingly similar to our case, confirming the assumption that the lesions seen in our fulminant case belong to pattern II type demyelination.

## 4. Experimental Section

### 4.1. Patient Case History and Outcome

A 15-year-old male patient was admitted to the Department of Neurology, University of Szeged, Hungary, in November 2004, since two days after a Hepatitis-B vaccination his right limbs became weak and developed movement coordination problems. His disease started in March 2004, when he was admitted to a pediatric department because of dizziness and vomiting. During his observation muscle weakness developed on his right extremities, with vestibular and cerebellar symptoms. Brain magnetic resonance imaging (MRI) showed multiple contrast-enhancing white-matter lesions. In September 2004 he was admitted to hospital with left lower limb weakness and cerebellar symptoms. His symptoms improved.

#### 4.1.1. Examinations

**Figure 3:**
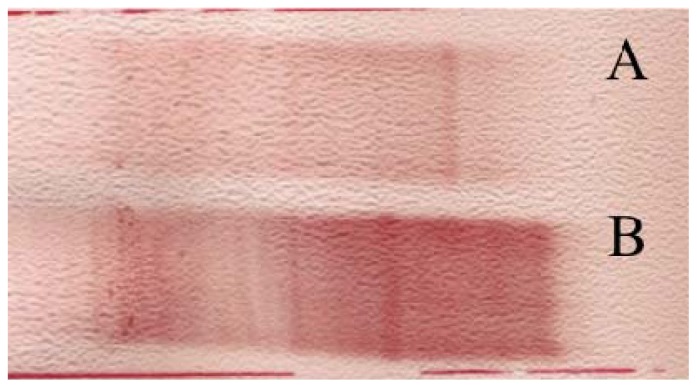
Immunoblot of CSF from a fulminant multiple sclerosis patient at two different time points; (**A**) (FM1) and (**B**) (FM2).

Cerebrospinal fluid (CSF) analysis showed a protein content of 0.57 g/L (determined by laser nephelometry), wbc (white blood cell count): 15 × 10^6^ cells/L, rbc (red blood cell count): 8 × 10^6^ cells/L. Albumin quotient (Q_alb_) of 7.7 indicated an intact blood brain barrier (BBB). The IgG index was elevated (0.74). The immunoblot was positive for IgG and oligoclonal IgG bands were found with isoelectric focusing (Figure 3).

#### 4.1.2. Electrophysiology

**Figure 4:**
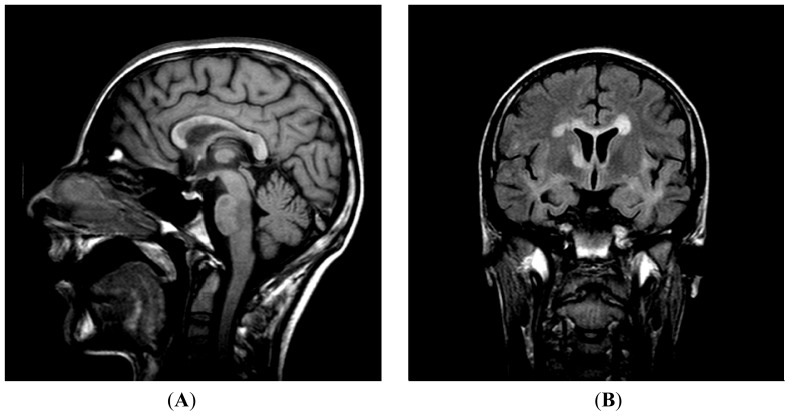
Brain MRI. (**A**) 2006 T1 Sagittal; (**B**) 2006 T2 Flair.

Somatosensory Evoked Potentials (SSEP) showed severe central conduction damage (increased central conduction times) and findings characteristic of brainstem and subcortical lesions. According to McDonald’s criteria [25] the patient was diagnosed as suffering from multiple sclerosis with relapsing-remitting clinical form. The patient had his fourth relapse in January 2005. He had 4 relapses in 1 year, so it was decided to put him on immunomodulatory treatment (interferon-β 1b sc., every other day). Despite the immunomodulatory treatment he had 4 more relapses in 2005 and a continuous progression of the disease was observed. The EDSS score (Expanded Disability Status Scale [26]) was 3.5 points. A second brain MRI was performed. Compared to the first MRI the number and size of lesions increased. There were lesions in the pons, cerebellum on both sides, frontal and temporal white matter, parietal subcortical region and capsula interna (Figure 4A,B). In 2006 after recurrent urinary infections the patient became unable to walk. On admission neurological examination showed ptosis on the right, internuclear ophtalmoparesis and tetraparesis. He had a permanent catheter due to urine retention. The EDSS score was 8 points. On the 6th day of observation the patient became cyanotic and had periodic breathing. He was intubated and transferred to the Intensive Care Unit (ICU). The second lumbar puncture (LP) was performed to exclude meningitis. The protein content was 0.68 g/L, wbc: 80× 10^6^ cells/L, 95% lymphocytes (ly), 5% monocytes (mo), rbc: 810× 10^6^ cells/L. The albumin index was elevated (12.9), indicating a BBB (blood brain barrier) damage. The IgG index was also highly elevated, 1.88. Oligoclonal bands were found (Figure 3B). Results showed inflammation and increased cell count, but microbiological examinations of the CSF excluded meningitis. The disease spread to the hypothalamus: he had polyuria with 18 L/day urine volume. Because of recurring infections of the airways and urinary tract he was treated with antibiotics based on microbiological examinations. After a one month treatment in the ICU the patient died. Urosepsis or candida sepsis was presumed as the immediate cause of death.

#### 4.1.3. Pathology

**Figure 5:**
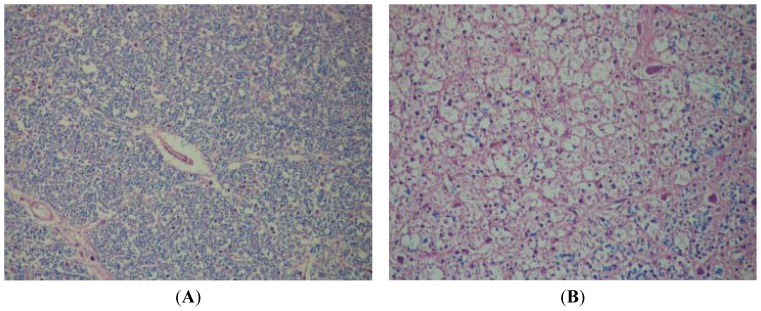
Brainstem: a luxol fast blue violet stain shows loss of myelin (**B**) in comparison to a normal myelinated area (**A**). A: magnification 10×; B: magnification 20×.

In coronal sections of the formalin fixed brain and spinal cord no specific macroscopic features were observed including plaques or atrophy. Histologically widespread myelin loss was observed in the brainstem and optic chiasm. Widespread perivascular and scattered interstitial lymphocytes were also detected. To detect the myelin loss luxol fast blue staining was used (Figure 5A,B). Furthermore, perivascular and scattered interstitial lymphocytes were also observed using lymphocyte PAN T, B markers (CD3, CD20). Considering the clinical features and pathological findings the diagnosis of multiple sclerosis was made. The cause of death was severe bronchopneumonia.

### 4.2. Sampling

CSF samples were obtained from diagnostic lumbar puncture at the Department of Neurology, University of Szeged, Hungary. Samples were immediately frozen, transported on dry ice to the Department of Physical and Analytical Chemistry, Analytical Chemistry, Uppsala University, Sweden, and stored at −80 °C until analysis. CSF samples from a fulminant case of multiple sclerosis taken at two different time points were analyzed: at the time of diagnosis (FM1) and later at the time of rapid progression of the disease (FM2). We chose a CSF sample from a patient with relapsing-remitting (RR) clinical form of the disease (disease control) who has an EDSS score of only 2 points after 20 years duration of the disease, indicating a good functional status, and a CSF sample from a headache patient whose diagnostic CSF analysis proved to be normal (healthy control). Table 2 shows demographic and clinical data of the multiple sclerosis and control subjects.

### 4.3. Ethics

The study was approved by the Human Investigation Review Board of the University of Szeged, Albert Szent-Györgyi Clinical Centre and it agrees with the Declaration of Helsinki (Ethical permission # 93/2007).

### 4.4. Chemicals and Reagents

Acetonitrile (ACN), methanol (MeOH), acetic acid (HAc), ammonium-dihydrogen-phosphate, phosphoric acid and sodium hydroxide were obtained from Merck (Darmstadt, Germany). Urea and trifluoroacetic acid (TFA) were purchased from Sigma Aldrich (St. Louis, MO, USA). High purity water was taken from a Milli-Q (Millipore, Bedford, MA, USA) purification system.

### 4.5. Protein Digestion and Isobaric Tag Labeling Procedure

Four samples were chosen for quantitative protein analysis by isobaric tag labeling using the iTRAQ 4plex kit (Applied Biosystems, Foster City, CA, USA). The procedure was followed according to the manufacturers’ instructions. The protocol is described in detail elsewhere [27]. Briefly, for each sample, a volume corresponding to 100 μg total protein (as determined by Bradford protein assay) was aliquoted. The normalized samples were individually subjected to reduction and alkylation prior enzymatic digestion for 24 h with trypsin. Each sample was subsequently labeled with iTRAQ 114–117 and samples were combined for multiplexed LC MS/MS analysis.

### 4.6. Liquid Chromatography–Mass Spectrometry

Nanoscale liquid chromatography coupled to MALDI mass spectrometry experiments were performed as described previously [27]. Briefly, an amount of 1.5 μg labelled and combined protein digestion products were analyzed using a 1100 nanoflow LC system equipped with a micro fraction collector (Agilent Technologies, Waldbronn, Germany). Peptide gradient elution was followed by direct fractionation onto a MALDI target plate (disposable PAC target, Bruker Daltonics, Bremen Germany) resulting in 384 fractions. The targets were washed with 10 mM NH_4_H_2_PO_4_/0.1% TFA after sample application and before subjection to the mass spectrometer. The detailed protocol for mass spectrometry based protein identification is described in detail elsewhere [8]. Briefly, peptide mass data were acquired with an Ultraflex II MALDI-TOF/TOF MS (Bruker Daltonics, Bremen, Germany) in reflector positive mode. Data acquisition was assisted by the WarpLC Software (Bruker Daltonics) for automatic TOF-MS spectra acquisition, background signal filtering, grouping of intact peptide signals into a peptide profile with respect to their distribution and intensity as well as optimized precursor ion selection for subsequent MS/MS experiments. For final protein identification all MS/MS data were analyzed in a comprehensive MS/MS ion search using the Mascot search engine (v 2.2, Matrix Science, London, UK). The relative abundance of proteins in the 3 MS samples (FM1, FM2, RR) was expressed as ratios compared to the control sample. The measurement was repeated to show reproducibility of the method. A difference in abundance of 0.5 or above was determined to be significant prior to data analysis. The difference in relative abundance of >0.5 was marked with “+”, more than 1.0 with “++” and more than 1.5 with “+++” [28].

## 5. Conclusions

Our study found a number of proteins that may have a role in rapid disease progression in multiple sclerosis. Further studies are needed to relate the function of these proteins to disease pathology. Current diagnostic CSF tests examine the presence of oligoclonal bands and IgG index to support the diagnosis of MS [29]. Proteomic studies of CSF may lead to the development of more specific biomarkers that will allow earlier diagnosis and distinction between clinical forms of the disease. The search for biomarkers that are able to identify patients at high risk for rapid progression becomes increasingly important with the appearance of more aggressive treatment possibilities. The shortcoming of our study is the small sample size. In another ongoing study we currently analyze LC-FTICR MS [30–32] data of a larger set of CSF samples from a variety of clinical forms of MS and matched controls.

Despite the increasing number of studies investigating potential biomarkers of MS disease progression and response to therapy, there is still no protein that is repeatedly identified by different groups. This may be due to the relatively small sample sizes and the heterogeneity of the methods applied. Large scale multi-center projects using standard methods for collecting, storing and analyzing the samples are necessary to validate these preliminary results and integrate candidate biomarkers into the pathomechanism of the disease.

## Figures and Tables

**Table 1:** List of proteins significantly up-regulated in at least one of the multiple sclerosis cerebrospinal fluid (CSF) samples compared to control. The relative abundance values are shown for RR (relapsing-remitting), FM1 (fulminant MS time point 1) and FM2 (fulminant MS time point 2) CSF samples compared to control (C), which is considered 1. + difference > 0.5; ++ difference > 1.0; +++ difference > 1.5.

#	Protein Name	Database Entry a	Mascot score b	Number of Pep. c	MW d	Function	C	RR	Δ	FM1	Δ	FM2	Δ
	**Transport proteins**												
1	Serotransferrin	TRFE_HUMAN	1053	47	87.3411	Iron transport	1	1.171		**1.61**	**+**	1.16	
2	Apolipoprotein E	APOE_HUMAN	560	26	38.2412	Lipid transport	1	1.462		**1.86**	**+**	1.24	
3	Apolipoprotein A-IV	APOA4_HUMAN	37	6	49.5504	Lipid metabolism	1	**1.52**	**+**	1.31		**1.98**	**+**
4	Phospholipid transfer protein	PLTP_HUMAN	46	3	57.4823	Lipid transport	1	**1.611**	**+**	**1.75**	**+**	1.28	
	**Immunoglobulins**												
5	Ig gamma-3 chain C region	IGHG3_HUMAN	51	6	36.36	Immune response	1	**1.544**	**+**	1.47		**1.61**	**+**
6	Ig kappa chain C region	KAC_HUMAN	464	8	13.0366	Immune response	1	1.38		**1.63**	**+**	**2.56**	**+++**
7	Ig kappa chain V-I region	KV108_HUMAN	74	4	12.3321	Immune response	1	0.992		1.27		**1.54**	**+**
8	Ig lambda chain C regions	LAC_HUMAN	170	5	12.6644	Immune response	1	**1.572**	**+**	1.31		**2.02**	**++**
9	Ig gamma-1 chain C region	IGHG1_HUMAN	210	9	40.676	Immune response	1	1.426		**1.55**	**+**	**1.64**	**+**
10	Ig mu chain C region	MUC_HUMAN	233	11	53.3918	Immune response	1	**1.848**	**+**	**1.65**	**+**	**1.83**	**+**
11	Ig heavy chain V-III region TUR	HV318_HUMAN	54	3	13.0917	Immune response	1	**2.114**	**++**	**1.52**	**+**	**1.58**	**+**
	**Glycoproteins**												
12	Osteopontin	OSTP_HUMAN	462	7	38.4212	Cell communication	1	**1.644**	**+**	1.49		**3.39**	**+++**
13	Vitronectin	VTNC_HUMAN	28	4	57.9411	Cell communication	1	**1.574**	**+**	**1.78**	**+**	1.38	
14	Zinc-alpha-2-glycoprotein precursor	ZA2G_HUMAN	47	2	37.061	Cell adhesion. lipid degradation	1	**2.024**	**++**	**1.51**	**+**	**1.53**	**+**
15	Beta-2-glycoprotein 1	APOH_HUMAN	47	2	43.7975	Regulation of blood coagulation	1	1.341		**1.53**	**+**	**1.64**	**+**
16	SPARC-like protein 1 precursor	SPRL1_HUMAN	85	3	82.6318	Calcium ion binding	1	**1.677**	**+**	**1.82**	**+**	1.44	
17	Chitinase-3-like protein 1	CH3L1_HUMAN	78	5	46.0906	Tissue remodeling	1	1.334		**1.62**	**+**	1.38	
	**Coagulation factors**												
18	Fibrinogen beta chain	FIBB_HUMAN	165	8	61.7759	Cell proliferation	1	1.416		**1.83**	**+**	**1.89**	**+**
19	Fibrinogen alpha chain	FIBA_HUMAN	156	8	101.853	Cell proliferation	1	1.073		0.97		**1.88**	**+**
	**Complement factors**												
20	Complement factor B	CFAB_HUMAN	28	4	94.3637	Immune response	1	**1.725**	**+**	**1.97**	**+**	1.26	
21	Complement C4-A	CO4A_HUMAN	339	27	205.178	Immune response	1	1.474		**1.68**	**+**	**1.63**	**+**
	**Enzymes**												
22	Elongation factor 2 kinase	EF2K_HUMAN	29	4	88.2004	Translational elongation	1	**1.642**	**+**	**2.35**	**++**	1.16	
23	Prostaglandin-H2 D-isomerase	PTGDS_HUMAN	216	11	22.7844	Enzyme activity	1	1.178		**1.59**	**+**	1.03	
24	Cystatin-C	CYTC_HUMAN	249	12	17.1259	Enzyme regulator	1	**1.53**	**+**	**2.11**	**++**	**1.57**	**+**
	**Structural/membrane asssociated**												
25	Calsyntenin-1	CSTN1_HUMAN	90	3	117.941	Signal transduction	1	**1.596**	**+**	**2.21**	**++**	**1.62**	**+**
26	Limbic system-associated membrane protein precursor	LSAMP_HUMAN	73	1	40.3779	Cell adhesion. neuronal growth	1	**2.205**	**++**	**2.37**	**++**	**1.79**	**+**
27	Basement membrane-specific heparan sulfate proteoglycan core protein	PGBM_HUMAN	39	5	487.116	Protein binding	1	**2.014**	**++**	**2.8**	**+++**	**2.06**	**++**
28	Neural cell adhesion molecule 1.	NCA11_HUMAN	71	2	102.35	Cell adhesion	1	1.451		**1.77**	**+**	**1.53**	**+**
29	Monocyte differentiation antigen CD14	CD14_HUMAN	27	5	41.9976	Immune response	1	**1.569**	**+**	**1.846**	**+**	**1.88**	**+**
	**Miscellaneous**												
30	Serum amyloid A protein	SAA_HUMAN	87	2	14.4341	Acute phase response	1	1.27		**1.59**	**+**	**2.58**	**+++**

a Uniprot knowledgebase entry;

b Mascot protein score revealed by MudPIT scoring. Proteins were found and identified by integrated mascot database batch search of all MS/MS in Swissprot v 51.6. All matches are identified significantly. Identified proteins are considered as positive match on at least a 99% significance level (*p* < 0.01) corresponding to a significance threshold ionscore of 34;

c Number of tryptically peptides that match the identified protein. At least one matching peptide for each identified protein must fulfil criteria of significance (*p* < 0.01) and uniqueness;

d Molecular weight in kDa.

**Table 2:** Demographical and clinical data of multiple sclerosis patients and controls.

	Age (years)	Gender	Duration of the disease	EDSS
Fulminant MS t1	15	M	8 months	5
Fulminant MS t2	16	M	22 months	8
RR MS	25	F	20 years	2
Control	23	M	NA	NA

RR = relapsing-remitting, t1 = 1st lumbar puncture, t2 = 2nd lumbar puncture, M = male, F = female, EDSS = Expanded Disability Status Scale [26], NA = not applicable.
